# Addition of docetaxel to S-1 without platinum prolongs survival of patients with advanced gastric cancer: a randomized study (START)

**DOI:** 10.1007/s00432-013-1563-5

**Published:** 2013-12-24

**Authors:** Wasaburo Koizumi, Yeul Hong Kim, Masashi Fujii, Hoon Kyo Kim, Hiroshi Imamura, Kyung Hee Lee, Takuo Hara, Hyun Cheol Chung, Taroh Satoh, Jae Yong Cho, Hisashi Hosaka, Akihito Tsuji, Akinori Takagane, Mikito Inokuchi, Kazuaki Tanabe, Tatsuya Okuno, Mariko Ogura, Kazuhiro Yoshida, Masahiro Takeuchi, Toshifusa Nakajima, The JACCRO and KCSG Study Group

**Affiliations:** 1 Kitasato University School of Medicine, Sagamihara, Japan; 2 Korea University Anam Hospital, Seoul, South Korea; 3 Department of Digestive Surgery, Nihon University School of Medicine, 1-8-13, Kandasurugadai, Chiyoda-ku, Tokyo, Japan; 4 St. Vincent’s Hospital, Suwon, South Korea; 5 Sakai Municipal Hospital, Osaka, Japan; 6 Yeungnam University Hospital, Daegu, Korea; 7 Kouseiren Takaoka Hospital, Takaoka, Japan; 8 Yonsei University College of Medicine, Seoul, South Korea; 9 Osaka University School of Medicine, Osaka, Japan; 10 Gunma Prefectural Cancer Center, Gunma, Japan; 11 Kobe City Medical Center General Hospital, Kobe, Japan; 12 Hakodate Goryoukaku Hospital, Hakodate, Japan; 13 Tokyo Medical and Dental University, Tokyo, Japan; 14 Hiroshima University Hospital, Hiroshima, Japan; 15 Kobe University School of Medicine, Kobe, Japan; 16 Cancer Institute Hospital, Tokyo, Japan; 17 Gifu University School of Medicine, Gifu, Japan; 18 Kitasato University School of Pharmacy, Tokyo, Japan; 19 Japan Clinical Cancer Research Organization, Tokyo, Japan

**Keywords:** Advanced gastric cancer, Chemotherapy, S-1, Docetaxel

## Abstract

**Purpose:**

Cisplatin plus 5-fluorouracil has been globally accepted as a standard regimen for the treatment for advanced gastric cancer. However, cisplatin has several disadvantages, including renal toxicity and the need for admission. S-1 plus cisplatin has become a standard treatment for advanced gastric cancer in East Asia. This phase III study was designed to evaluate the potential benefits of adding docetaxel to S-1 without a platinum compound in patients with advanced gastric cancer.

**Methods:**

Patients were randomly assigned to receive docetaxel plus S-1 or S-1 alone. The docetaxel plus S-1 group received docetaxel on day 1 and oral S-1 on days 1–14 of a 21-day cycle. The S-1 alone group received oral S-1 on days 1–28 of a 42-day cycle. The primary end point was overall survival.

**Results:**

Of the 639 patients enrolled, 635 were eligible for analysis. The median overall survival was 12.5 months in the docetaxel plus S-1 group and 10.8 months in the S-1 alone group (*p* = 0.032). The median progression-free survival was 5.3 months in the docetaxel plus S-1 group and 4.2 months in the S-1 alone group (*p* = 0.001). As for adverse events, neutropenia was more frequent in the docetaxel plus S-1 group, but remained manageable.

**Conclusion:**

As first-line treatment for advanced gastric cancer, docetaxel plus S-1 significantly improves median overall and progression-free survival as compared with S-1 alone. (ClinicalTrials.gov number: NCT00287768).

## Introduction

Gastric cancer is the second most common cause of cancer death worldwide. The only potentially curative treatment for patients with gastric cancer is surgical resection. However, regional and distant recurrence often occurs after surgery. The standard treatment for advanced or recurrent gastric cancer is chemotherapy, hoping to prolong survival.

In the late 1990s, cisplatin plus 5-fluorouracil was globally accepted as a benchmark treatment for advanced gastric cancer and has since been used in many controlled clinical trials of new chemotherapeutic regimens (Ohtsu et al. 2003; Van Cutsem et al. 2006; Al-Batran et al. 2008; Cunningham et al. 2008; Boku et al. 2009). However, cisplatin has several important drawbacks, including high incidences of nausea, vomiting (Kris et al. 2006), and renal toxicity (Fillastre and Raguenez-Viotte 1989; Arany and Safirstein 2003) the need for admission to receive therapy, and other adverse events negatively affecting the quality of life of patients. Cisplatin is contraindicated in patients with poor renal function. The development of combination chemotherapy regimens that do not include platinum compounds has thus been awaited as a new option for the first-line treatment for advanced gastric cancer.

During the planning phase of this trial, docetaxel (Mai et al. 1999; Graziano et al. 2000; Mavroudis et al. 2000; Bang et al. 2002) and S-1, an oral preparation combining tegafur (a prodrug of fluorouracil) with gimeracil and oteracil potassium in a molar ratio of 1:0.4:1, were shown to be effective as monotherapy against advanced gastric cancer (Sakata et al. 1998; Shirasaka 2009). In Western countries, a phase III study comparing docetaxel plus cisplatin/5-fluorouracil with cisplatin plus 5-fluorouracil was ongoing in patients with advanced gastric cancer (V325 study). In Japan, S-1 had become the most widely used drug for the treatment for advanced gastric cancer, and phase III studies of S-1 plus cisplatin versus S-1 alone (SPIRITS trial: Koizumi et al. 2008) and S-1 plus irinotecan versus S-1 alone (TOP-002 trial: Narahara et al. 2011) were ongoing.

Phase II and phase I/II studies of S-1 plus docetaxel in Japanese patients with advanced gastric cancer have reported response rates of 56.2 and 45.7 %, with median survival times of 14.3 and 14.0 months, respectively (Yoshida et al. 2006; Yamaguchi et al. 2006). Although the dose of docetaxel (40 mg per square meter of body surface area) was lower than that used in Western countries, treatment was well tolerated in both studies, suggesting that S-1 plus docetaxel is a promising new regimen for the chemotherapeutic management of advanced gastric cancer. To confirm and extend these results, we performed a controlled study comparing S-1 plus docetaxel with S-1 alone as first-line chemotherapy for advanced gastric cancer, without the use of any platinum compounds.

## Patients and methods

The Japan Clinical Cancer Research Organization (JACCRO) GC-03 study (START trial) was a multicenter, prospective, randomized, phase III open-label trial performed by the JACCRO and the Korean Cancer Study Group (KCSG) ("Appendix"). Patients were registered and followed up using the FLADS^®^ system (Takt Systems, Inc., Tokyo, Japan), a Web-based registration and data collection system for clinical trials of anticancer therapy.

This study was performed in accordance with the declaration of Helsinki and the ethical guidelines and regulations of each country. The protocol was approved by the ethics committees of each center before the initiation of enrollment. An independent Response and Safety Evaluation Committee reviewed all efficacy and safety data.

Written informed consent was obtained from all patients before enrollment in the study. Eligible patients had to satisfy the following criteria: an expected survival of 3 months or longer; no prior chemotherapy; an age of 20 to younger than 80 years; an Eastern Cooperative Oncology Group performance status of zero or one; a histologically confirmed diagnosis of unresectable or advanced gastric adenocarcinoma (including adenocarcinoma of the gastroesophageal junction) or unresectable recurrence; ability to orally intake food and liquids; either measurable or non-measurable lesions as defined by the Response Evaluation Criteria in Solid Tumors (RECIST), version 1.0 (Therasse et al. 2000); and adequate organ functions.

Patients were excluded if they had any of the following conditions: another active cancer; severe ascites requiring drainage; grade 2 or severer peripheral neuropathy, pulmonary fibrosis, or interstitial pneumonitis; or a history of chemotherapy or radiotherapy for advanced gastric cancer.

### Treatment and testing

Patients were stratified according to institutions and whether they had measurable or non-measurable lesions and were then randomly assigned to receive docetaxel plus S-1 or S-1 alone. Treatment was continued until the onset of progressive disease, the development of toxicity meeting the criteria for drug withdrawal, or the withdrawal of consent by the patient. The docetaxel plus S-1 group received docetaxel (40 mg per square meter of body surface area) as a 1-h intravenous infusion on day 1 and oral S-1 on days 1–14 of a 21-day cycle. The daily dose of S-1 (given in two divided doses) was assigned according to body surface area as follows: <1.25 m^2^, 80 mg daily; ≥1.25–<1.5 m^2^, 100 mg daily; and ≥1.5 m^2^, 120 mg daily. The S-1 alone group received the same dose levels of S-1 as the docetaxel plus S-1 group, similarly assigned according to body surface area, on days 1–28 of a 42-day cycle. In the event of predefined toxic events, protocol-specified treatment modifications were permitted. Adverse events were graded according to the National Cancer Institute Common Toxicity Criteria for Adverse Events (NCI-CTCAE), version 3.0.

### Statistical analysis

The primary end point was overall survival, defined as the interval between enrollment and death from any cause. Secondary end points were progression-free survival, response rate, and safety. Response rates were based on the assessment of response by the investigators at each center and were reviewed by the Central Review Board of JACCRO; response was evaluated in accordance with RECIST, version 1.0.

**Fig. 1:**
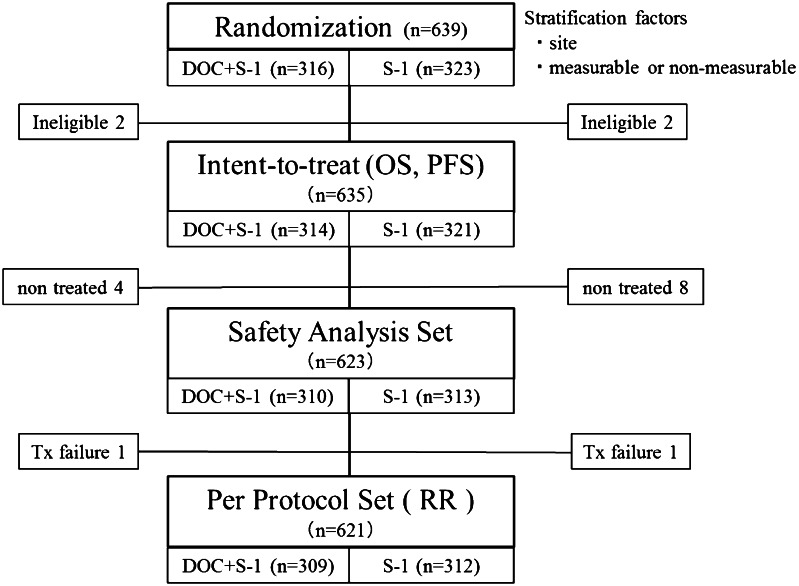
CONSORT diagram. *OS* overall survival, *PFS* progression-free survival, *RR* response rate

The overall survival and progression-free survival were calculated on the basis of an intent-to-treat analysis, and response rates were calculated on the basis of the per-protocol set (Fig. 1). Survival curves were calculated by the Kaplan–Meier method, and differences between groups were compared with the use of log-rank tests. In two previous phase II studies, the median survival times of patients with previously untreated advanced gastric cancer who received S-1 alone were 250 and 207 days (Sakata et al. 1998; Koizumi et al. 2000), respectively. Two phase II studies of docetaxel plus S-1 reported median survival times of 434 and 427 days, respectively (Yoshida et al. 2006; Yamaguchi et al. 2006). On the basis of these results, the median survival time was assumed to be 400 days in the docetaxel plus S-1 group and 300 days in the S-1 alone group. We estimated that a total enrollment of 628 patients was needed for the study to have a 90 % power to detect a difference in overall survival between the treatment groups with a two-sided alpha value of 0.05, assuming an enrollment period of 3 years, a follow-up period of 2 years, and a 10 % exclusion rate due to ineligibility. We planned an interim analysis after 377 deaths had been confirmed to decide whether to terminate the study.

This study was registered with ClinicalTrials.gov (ClinicalTrials.gov Identifier: NCT00287768).

### Role of the funding source

JACCRO and KCSG employees contributed to the study design and data collection and interpretation. This study was supported by an unconditioned grant from Sanofi K.K. Japan. Sanofi K.K. Japan had no role in the study design, data collection, analysis, or interpretation, or in writing the manuscript or deciding whether it would be submitted for publication. Masahiro Takeuchi and Masashi Fujii had access to the raw data. The corresponding author had full access to all study data and final responsibility for the decision to submit for publication.

## Results

### Patients’ characteristics

Between September 2005 and September 2008, a total of 113 centers participated (97 in Japan and 16 in Korea), and a total of 639 patients were registered by means of the FLADS^®^ system; 316 were assigned to the docetaxel plus S-1 group, and 323 were assigned to the S-1 alone group.

Four patients were ineligible because they did not have measurable or non-measurable lesions as defined by RECIST: two assigned to the docetaxel plus S-1 group and two assigned to the S-1 alone group. The intent-to-treat analysis thus included 635 patients, 314 in the docetaxel plus S-1 group and 321 in the S-1 alone group (Fig. 1). The baseline characteristics of the patients were similar in the two treatment groups (Table 1).

**Table 1:** Baseline patient characteristics

Characteristics	DOC+S-1 (*n* = 314)	S-1 (*n* = 321)	*p* value
Sex			
Male	227	229	0.79
Female	87	92	
Age (years)			
Median	65	65	0.66
Range	23–79	27–79	
ECOG PS			
0	137	147	0.58
1	177	174	
Primary lesion			
−	168	163	0.49
+	146	158	
Measurable lesions			
−	72	72	0.88
+	242	249	
Diagnosis			
Advanced	260	267	0.90
Relapse	54	54	
Adjuvant chemotherapy			
−	292	297	0.82
+	22	24	
No. of organs involved			
1	96	87	0.43
2	116	115	
≥3	102	119	
Metastasis			
Lymph nodes	215	225	0.66
Liver	108	107	0.78
Lung	19	28	0.20
Bone	9	12	0.54
Peritoneum	119	131	0.45

*ECOG PS* Eastern Cooperative Oncology Group performance status

### Efficacy

**Fig. 2:**
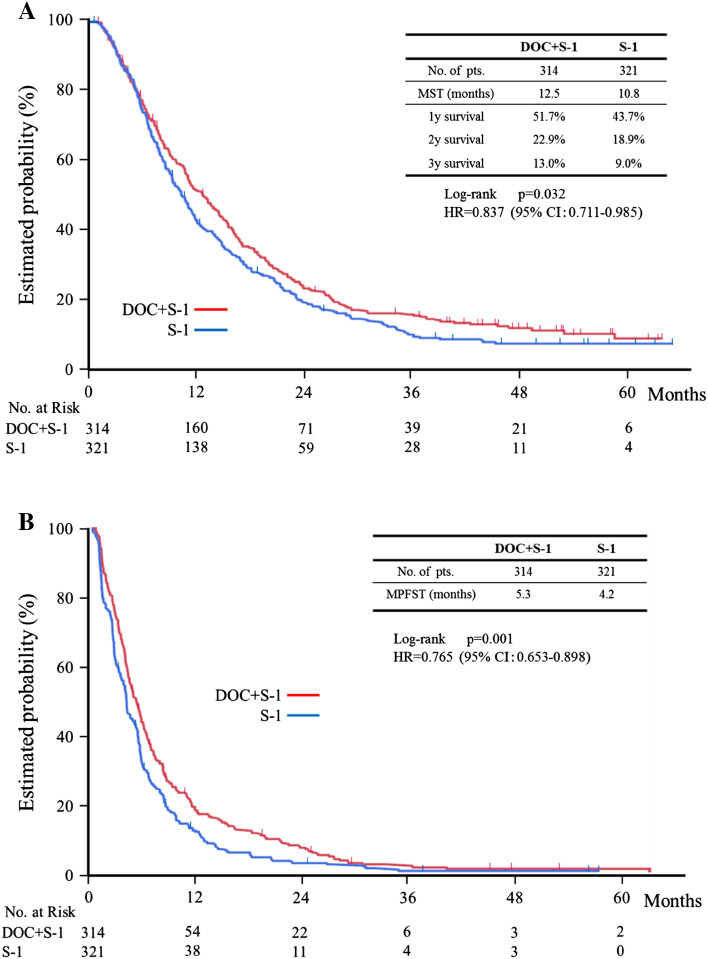
Kaplan–Meier estimate of overall survival and progression-free survival. **a** Overall survival. **b** Progression-free survival

Median follow-up of intent-to-treat group (ITT) was 11.4 months (inter-quartile range 6.21–21.2). Of 635 cases, 582 died, 36 were still alive, and 17 were lost to follow-up. The median overall survival time was 12.5 months (95 % confidence interval (CI) 11.4–14.8) in the docetaxel plus S-1 group and 10.8 months (95 % CI 9.5–11.8) in the S-1 alone group. This difference in overall survival was statistically significant (*p* = 0.032; hazard ratio (HR) 0.84; 95 % CI 0.71–0.99) (Fig. 2a). Progression-free survival also differed significantly and was 5.3 months (95 % CI 4.5–5.9) in the docetaxel plus S-1 group and 4.2 months in the S-1 alone group (95 % CI 3.7–4.7; *p* < 0.001; HR 0.77; 95 % CI 0.65–0.90) (Fig. 2b). The response rate was calculated on the basis of the 480 (77.3 %) of the 621 patients in the per-protocol set. The response rate was 38.8 % (95 % CI 32.8–45.2; complete response, 3; partial response, 89) among the 237 patients with measurable lesions in the docetaxel plus S-1 group and 26.8 % (95 % CI 21.6–32.6; complete response, 5; partial response, 60) among the 243 patients with measurable lesions in the S-1 alone group. This difference was statistically significant (*p* = 0.005).

**Fig. 3:**
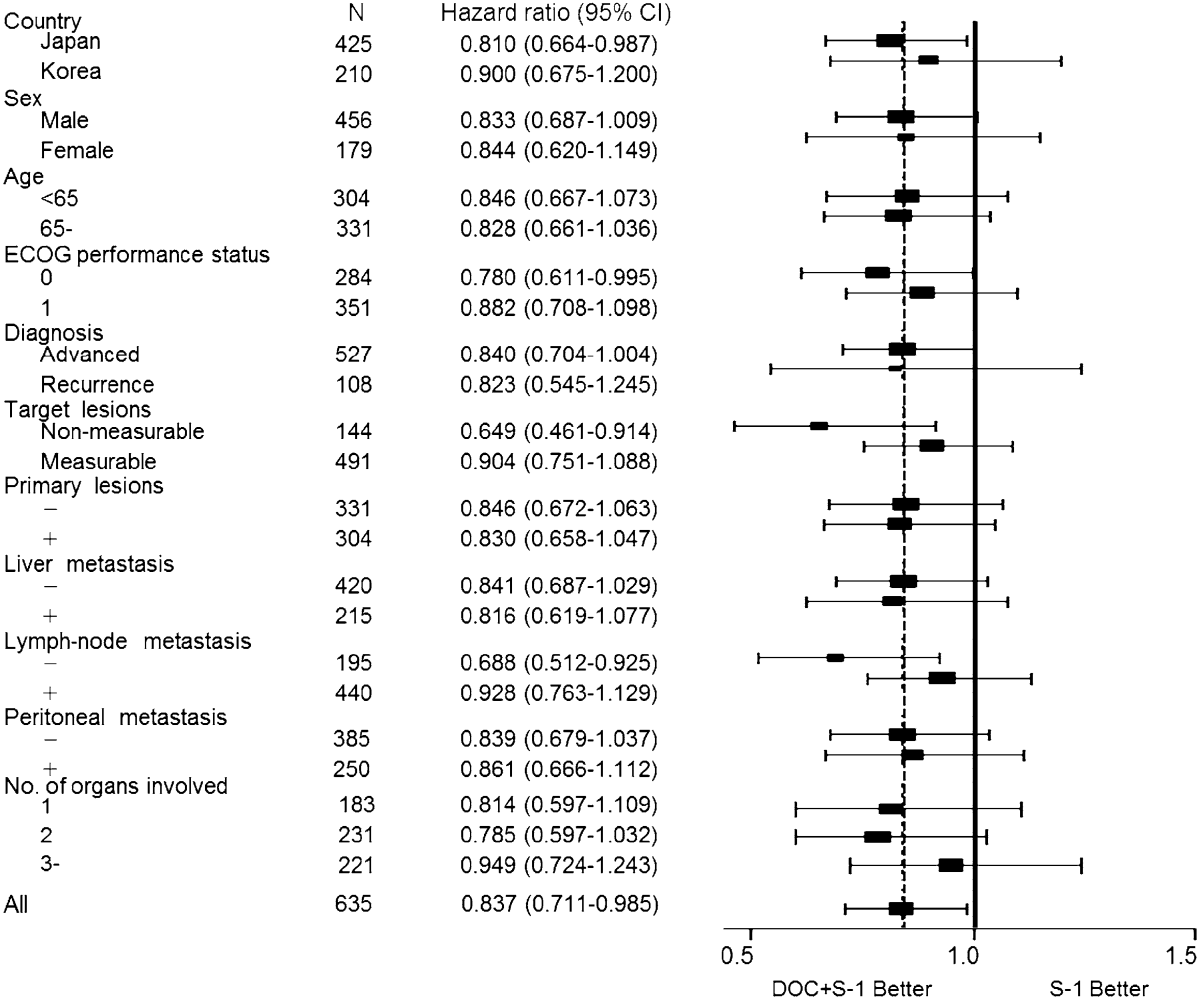
Forest plot of the treatment effect on overall survival in subgroup analysis

**Fig. 4:**
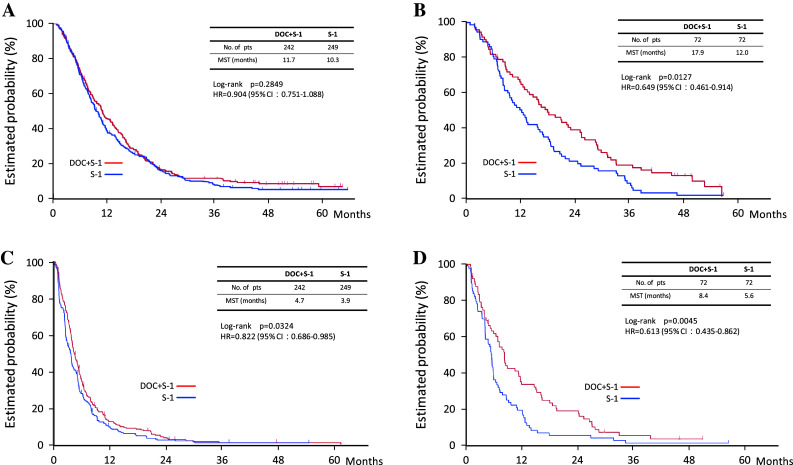
Overall survival and progression-free survival in subgroup analysis. **a** Overall survival in the measurable population. **b** Overall survival in the non-measurable population. **c** Progression-free survival in the measurable population. **d** Progression-free survival in the non-measurable population

On subgroup analysis, docetaxel plus S-1 showed significantly better overall survival than S-1 alone in patients with a performance status of zero, patients with non-measurable lesions, patients with no lymph-node metastasis, and Japanese patients than in patients with performance status one, patients with measurable lesions, patients with lymph-node metastasis, and Korean patients (Figs. 3, 4). Peritoneal metastasis was found in 109 (76 %) of the 144 patients with non-measurable lesions.

### Treatment and compliance

The median relative dose intensity was 80.4 % for docetaxel and 76.0 % for S-1 in the docetaxel plus S-1 group and 76.2 % for S-1 in the S-1 alone group. Treatment was delayed in 14.5 % of the patients in the docetaxel plus S-1 group and 4.2 % of those in the S-1 alone group. The main reason for treatment delays was adverse events in both groups. The main reason for withdrawal of treatment was disease progression in both groups. After withdrawal of the protocol treatment, second-line therapy was given to 69.7 % of the patients in the docetaxel plus S-1 group and 76.0 % of those in the S-1 group.

Among the Japanese patients who received second-line chemotherapy, 79 % were given irinotecan, cisplatin, or taxanes, while most of the 60 % of Korean patients who received second-line chemotherapy were given 5-fluorouracil-based regimens.

### Safety

The incidence of grade 3 or higher adverse events was 58.1 % in the docetaxel plus S-1 group and 39.6 % in the S-1 alone group (*p* < 0.0001). The incidences of grade 3 or higher leukopenia and neutropenia were significantly higher in the docetaxel plus S-1 group than in the S-1 alone group (*p* < 0.0001) and that of febrile neutropenia was significantly higher in the docetaxel plus S-1 group than in the S-1 alone group (*p* = 0.0024; Table 2). There were two treatment-related deaths in the docetaxel plus S-1 group (0.6 %).

**Table 2:** Safety (adverse events)

Adverse event	DOC+S-1 (*n* = 310)		S-1 (*n* = 313)		*p* value
	≥Grade 3	(%)	≥Grade 3	(%)	
Any	180	(58.1)	124	(39.6)	<0.001
Leukopenia	68	(21.9)	8	(2.6)	<0.001
Neutropenia	90*	(29.0)	14	(4.5)	<0.001
Platelets	5*	(1.6)	2	(0.6)	0.249
Hemoglobin	36	(11.6)	25	(8.0)	0.128
AST	3	(1.0)	7	(2.2)	0.208
ALT	3	(1.0)	6	(1.9)	0.321
Bilirubin	4	(1.3)	8	(2.6)	0.251
Creatinine	1	(0.3)	2	(0.6)	0.568
Nausea	18	(5.8)	11	(3.5)	0.175
Vomiting	10	(3.2)	7	(2.2)	0.449
Diarrhea	9	(2.9)	15	(4.8)	0.221
Stomatitis	13	(4.2)	5	(1.6)	0.053
Anorexia	48	(15.5)	37	(11.8)	0.183
Fatigue	18	(5.8)	15	(4.8)	0.572
Rash	3	(1.0)	6	(1.9)	0.321
Febrile neutropenia	9	(2.9)	0	(0)	0.002

Asterisk indicates one treatment-related death in each adverse event

Adverse drug reactions are based on CTCAE V3.0

*AST* aspartate aminotransferase, *ALT* alanine aminotransferase

## Discussion

Several pivotal phase III clinical trials have been performed in patients with advanced gastric cancer, including the V325 study of docetaxel plus cisplatin/5-fluorouracil by the V325 Study Group (Van Cutsem et al. 2006), a randomized trial of epirubicin plus cisplatin/5-fluorouracil by Cunningham and co-workers (Webb et al. 1997; Waters et al. 1999; Ross et al. 2002), and the SPIRITS trial of S-1 plus cisplatin by the SPRITS Trial Group (Koizumi et al. 2008). These trials have established the aforementioned regimens as standard treatments in their respective regions. All of these first-line regimens contain cisplatin. Our study in patients with advanced gastric cancer showed that adding docetaxel to S-1 significantly improved the overall survival, progression-free survival, and response rate as compared with S-1 alone. The survival benefit of docetaxel plus S-1 is particularly important, because regimens without platinum compounds would be a new option for the first-line treatment for advanced gastric cancer.

When we first reported the interim results of this trial at the American Society of Clinical Oncology (ASCO) Gastrointestinal Symposium 2012, the primary end point was not yet met. However, an independent statistician pointed out that data on more than 20 % of the patients had been censored and suggested that this problem should be solved to accurately evaluate treatment effectiveness. We therefore extended follow-up to 2 years after enrollment of the final patient, similar to the duration of follow-up at the initial analysis, to confirm the outcomes of the many patients with censored data. There was no alpha spending on reanalysis.

Docetaxel is a semisynthetic taxoid anticancer drug that is used as monotherapy or in combination with other anticancer agents to treat various cancers (Einzig et al. 1996; Chan et al. 1999; Shepherd et al. 2000; O’Shaughnessy et al. 2002; Fossella et al. 2003: Tannock et al. 2004; Martin et al. 2005; Marty et al. 2005; Posner et al. 2007). In the V325 study, docetaxel plus cisplatin/5-fluorouracil was compared with cisplatin/5-fluorouracil. The overall survival was significantly longer for docetaxel plus cisplatin/5-fluorouracil (9.2 months) than for cisplatin/5-fluorouracil (8.6 months; *p* = 0.02) (Van Cutsem et al. 2006). Although the doses of docetaxel differed between the V325 study (75 mg/m^2^) and our study (40 mg/m^2^), the benefit of adding docetaxel was confirmed in two large randomized studies of V325 and our study in advanced gastric cancer (Van Cutsem et al. 2006).

The main toxic effect of docetaxel is myelosuppression. In the V325 study, the incidence of severe neutropenia was 82 % in patients who received docetaxel plus cisplatin/5-fluorouracil, approved in Western countries for the treatment for advanced gastric cancer (Van Cutsem et al. 2006). Many modified regimens of docetaxel plus cisplatin/5-fluorouracil with better safety profiles can be used (Roth et al. 2007; Lorenzen et al. 2007; Tebbutt et al. 2010; Shah et al. 2010; Inal et al. 2012). In our study, we used docetaxel in a dose of 40 mg/m^2^, which was based on the results of Japanese phase I/II studies, and grade 3 or higher neutropenia occurred in only 29 % of the patients who received docetaxel plus S-1. Because the rate of severe neutropenia was low in our study, the dose of docetaxel might be able to be increased slightly, approaching the level used in Western countries, but since docetaxel plus S-1 therapy was administered on an outpatient basis, our dose setting might be appropriate for Asian patients and is considered effective in terms of survival.

The tumor burden is a more important prognostic factor than other factors, such as performance status or whether the patient receives adjuvant chemotherapy. To our knowledge, the present study is the first large randomized clinical trial to stratify patients with advanced gastric cancer according to measurable or non-measurable lesions. In our pre-planned subgroup analysis, there was no statistically significant difference in overall survival between the docetaxel plus S-1 group (11.7 months; *n* = 242) and the S-1 alone group (10.3 months; *n* = 249; HR 0.90; *p* = 0.28) among patients with measurable lesions. Among patients with non-measurable lesions, however, the overall survival was significantly longer in the docetaxel plus S-1 group (17.9 months; *n* = 72) than in the S-1 alone group (12.0 months; *n* = 72; HR 0.65; *p* = 0.013) (Fig. 3). In an exploratory subgroup analysis of the SPIRITS trial, S-1 plus cisplatin was associated with a higher benefit in patients with non-target tumors than in those with target tumors (Koizumi et al. 2008).

For pre-stratified measurable tumors, the overall survival benefit of docetaxel plus S-1 was not significant, but progression-free survival was significantly better with docetaxel plus S-1 (4.7 months) than with S-1 alone (3.9 months; HR 0.82; *p* = 0.03). This finding might be attributed to the notion that overall survival time is influenced by effective second-line chemotherapy, whereas progression-free survival is not. For patients with measurable tumors, more active regimens for combination chemotherapy, such as triplet regimens or regimens including new biological agents, should be investigated.

The present study was conducted in Japan and Korea, and the recommended dose of S-1 differs between Asian patients (80 mg/m^2^) and those in Western countries (50 mg/m^2^) (Ajani et al. 2010). Our results therefore cannot be extrapolated to all patients with gastric cancer. However, docetaxel plus S-1 therapy appears to be a promising, non-platinum-based treatment option for patients with gastric cancer in East Asia, which accounts for about 60 % of all cases of gastric cancer in the world. The results of our study may also provide important clues to the development of S-1-based, non-platinum regimens in Europe and North America.

In conclusion, docetaxel plus S-1 therapy is expected to become an important treatment option for patients with advanced gastric cancer, particularly those with compromised renal function or who want to receive chemotherapy on an outpatient basis.

## Appendix

Chief investigator: M. Fujii.

Members of the JACCRO and KCSG study teams were as follows:

The protocol design group: H. Baba, H. Takiuchi, K. Yoshida, T. Kubota, W. Koizumi; the efficacy and safety assessment committee: M. Takeuchi, T. Sasaki, Y. Shimada; statistical analyst: M. Takeuchi; principal investigators: (JACCRO group in Japan) Aichi Cancer Center—K. Muro, T. Ura; Aichi Medical University—K. Suzumura; Akita University Hospital—M. Odajima; Aomori Prefectural Central Hospital—S. Saitoh; Asahikawa Kosei Hospital—M. Goto; Asahikawa Medical University Hospital—K. Moriichi, T. Hoshi; Cancer Institute Hospital—K. Chin, M. Suenaga, Y. Kuboki; Dokkyo Medical University—K. Miyachi; Ehime University Hospital—Y. Kojima; Fukui Prefectural Hospital—O. Hosokawa; Fukui Red Cross Hospital—H. Fujii; Fukushima Medical University—Z. Sase; Fukuyama City Hospital—S. Asami; Gifu Municipal Hospital—H. Oshita; Gifu Prefectural General Medical Center—H. Kato; Gifu University School of Medicine—F. Sakashita, K. Yamaguchi; Gunma Prefectural Cancer Center—H. Hosaka, N. Haga; Gunma University Graduate School of Medicine—R. Aihara; Hakodate Goryokaku Hospital—A. Takagane; Hirosaki University Graduate School of Medicine—H. Kawasaki, J. Itoh, T. Takahata; Hiroshima City Asa Hospital—N. Hirabayashi; Hiroshima Red Cross Hospital and Atomic-bomb Survivors Hospital—M. Yamamoto; Hiroshima University—K. Tanabe; Hospital, University of the Ryukyus—M. Nakamoto; Hyogo College of Medicine—Y. Fujiwara; Ibi Kosei Hospital—S. Tachibana; Iizuka Hospital—T. Nagaie; International University of Health and Welfare Mita Hospital—K. Ohta; Iwate Medical University—K. Koeda; Jichi Medical University Hospital—M. Hyodo, Y. Hosoya; Jikei University School of Medicine—T. Sasaki; Kagoshima University Graduate School of Medical and Dental Sciences—A. Nakajo; Kanazawa Medical University—T. Kosaka; Kanazawa University—T. Fujimura; Kaneda Hospital—M. Uno; Keiju Medical Center—T. Kamata; Kinki Central Hospital—K. Kobayashi; Kinki University School of Medicine—M. Miyazaki, T. Sato, W. Okamoto; Kitasato University East Hospital—K. Higuchi, S. Tanabe, T. Sasaki, W. Koizumi; Kobe University Hospital—Y. Fujishima; Kochi Health Sciences Center—A. Tsuji; Kouseiren Takaoka Hospital—T. Hara; Kumamoto University Graduate School of Medical Sciences—M. Watanabe; Kurume University, School of Medicine—A. Keishiro; Kushiro City General Hospital—A. Goto; Kyorin University Hospital—O. Yanagida; Kyoto University Hospital—S. Matsumoto; Kyushu Central Hospital—K. Sumiyoshi; Kyusyu University Graduate School of Medicine—H. Saeki; Matsuyama Shimin Hospital—R. Watanabe, S. Yunoki; Minoh City Hospital—S. Iijima; Morioka Red Cross Hospital—Y. Sugimura; Nagano Municipal Hospital—K. Okita, Y. Munakata; Nagasaki University Graduate School of Biomedical Science—S. Hidaka; Nakadori General Hospital—H. Andoh; National Center for Global Health and Medicine—J. Akiyama; National Hospital Organization Hokkaido Cancer Center—Y. Takahashi; National Hospital Organization Kure Medical Center and Chugoku Cancer Center—N. Hatanaka; National Hospital Organization Nagasaki Medical Center—T. Nakata; National Hospital Organization Nagoya Medical Center—M. Kataoka; National Kyushu Cancer Center—H. Ariyama, T. Esaki; Nihon University School of Medicine—M. Kochi; Nippon Steel Yawata Memorial Hospital—R. Ohta; Osaka City University Graduate School of Medicine—B. Nakata; Osaka General Medical Center—K. Nishikawa; Rinku General Medical Center, Izumisano Municipal Hospital—H. Mizuno, M. Izukura, T. Mizushima; Saga University—M. Tanaka; Saiseikai Shiga Prefectural Hospital—T. Shigematsu; SaiseikaiYokohamashi Tobu Hospital—T. Egawa; Sakai Municipal Hospital—T. Kishimoto; Sapporo Medical University School of Medicine—T. Nobuoka; Shimane University School of Medicine—N. Hirahara; Showa University Fujigaoka Hospital—H. Nemoto; Showa University Hospital—K. Yamazaki, Y. Tajima; Social Insurance Chuo General Hospital, M. Shibasaki; St.Marianna University School of Medicine—T. Tsuda; St.Marianna University Yokohama-city Seibu Hospital—A. Hanai, K. Sumiyoshi, M. Kimura; Surugadai Nihon University Hospital—K. Hagiwara; Teikyo University School of Medicine—K. Iwasaki; Tohoku Rosai Hospital—H. Musha; Tohoku University—Y. Kakudo; Tokai University Hospital—K. Nabeshima; Toki Municipal General Hospital—T. Oiwa; Tokushima Red Cross Hospital—H. Ishikura, H. Okitsu; Tokyo Medical and Dental University—M. Inokuchi, S. Ooka; Tokyo Medical University—S. Hoshino; Tokyo Metropolitan Cancer and Infectious Diseases Center Komagome Hospital—Y. Iwasaki; Toranomon Hospital—T. Iizuka; Tottori University Faculty of Medicine—H. Saito, S. Tatebe; Toyonaka Municipal Hospital—J. Fujiita; University of Occupational and Environmental Health—A. Higure, K. Hirata; Yamagata Prefectural Central Hospital—H. Saito, K. Suzuki, T. Matsuda; Yamanashi Prefectural Central Hospital—M. Hada; Yokohama Municipal Citizen’s Hospital—M. Takahashi; (KCSG group in South Korea) Chungnam National University Hospital—S. Y. Kim; Department of Internal Medicine St. Mary’s Hospital—I. S. Woo; Hallym University Sacred Heart Hospital—D. Y. Zang; Hanyang University Guri Hospital—J. H. Choi; Inha University—M. H. Lee; Inje University Seoul Paik Hospital—H. G. Lee; Kangbuk Samsung Hospital—S. S. Lee; Korea University Anam Hospital—Y. H. Kim; Korea University Guro Hospital—J. S. Kim; Seoul National University Bundang Hospital—G. W. Lee; Seoul National University Hospital—T. Y. Kim; St. Vincent’s Hospital—H. K. Kim; Wonju Christian Hospital—J. I. Lee, K. Y. Shim; Yeungnam University Hospital—K. H. Lee; Yong Dong Severance Hospital—H. C. Jeung; Yonsei University College of Medicine—S. J. Kim.
